# Supplementary material for: Combined Inhibition of AKT and KIT Restores Expression of Programmed Cell Death 4 (PDCD4) in Gastrointestinal Stromal Tumor
Source: Cancers (Basel). 2021 Jul 23;13(15):3699. doi: 10.3390/cancers13153699 (PMC8345102; doi:10.3390/cancers13153699)
Supplement: Supplementary file 1 [file cancers-13-03699-s001.zip › Cancers-2021-PDCD4_in_GIST-Kozinova et al_Supplementary_figures_1_and_2.pdf]

Supplementary Materials

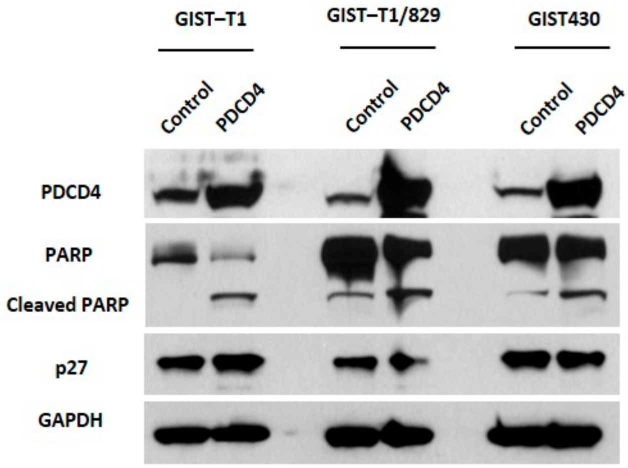


**Figure S1.** Overexpression of PDCD4 activates cleavage of PARP. Immunoblot assays of WCEs from GIST-T1, GIST-T1/829 and GIST430 after 72 hours following transfection. Equal quantities of WCE from each sample were subjected to immunoblotting with specific antibodies, as indicated. GAPDH served as loading control.

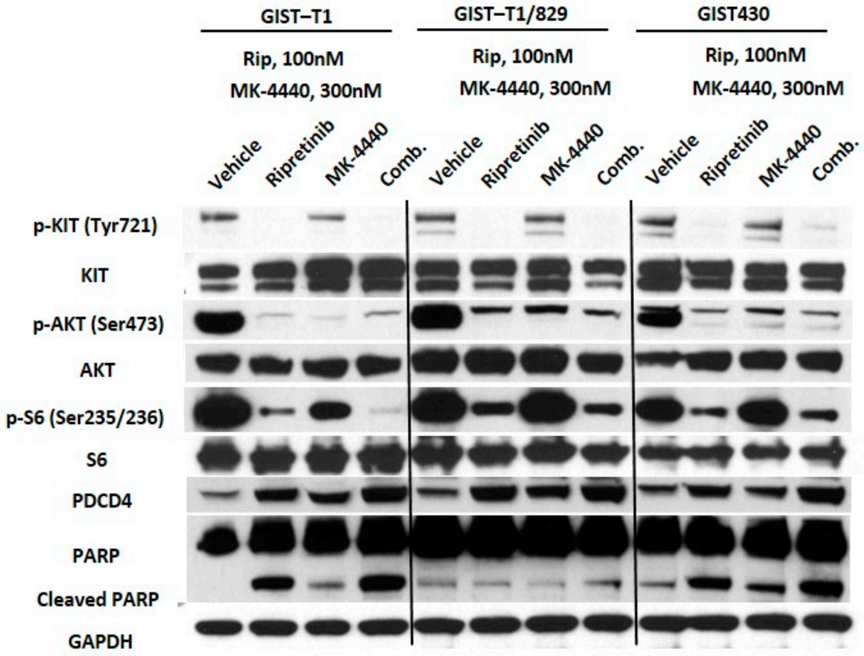


**Figure S2.** The combination of ripretinib and MK-4440 inhibits constitutive activation of effectors downstream of KIT. Immunoblot assays of WCEs from GIST-T1, GIST-T1/829 and GIST430 treated with 100nM Ripretinib, 300nM MK-4440 or the combination. All treatments were twenty hours in duration. Equal quantities of WCE from each sample were subjected to immunoblotting with specific antibodies, as indicated. GAPDH served as loading control.
