# Supplementary material for: Combined Inhibition of AKT and KIT Restores Expression of Programmed Cell Death 4 (PDCD4) in Gastrointestinal Stromal Tumor
Source: Cancers (Basel). 2021 Jul 23;13(15):3699. doi: 10.3390/cancers13153699 (PMC8345102; doi:10.3390/cancers13153699)
Supplement: Supplementary file 1 [file cancers-13-03699-s001.zip › Data file S3.pdf]

Supplementary data file S3: Immunoblots with molecular weight markers and densitometry

Figure 2

Samples' naming:

1 – GIST-T1 Veh

2 – GIST-T1 IM

3 – GIST-T1 MK-4440

4 – GIST-T1 Comb.

5 – GIST-T1/829 Veh

6 – GIST-T1/829 IM

7 – GIST-T1/829 MK-4440

8 – GIST-T1/829 Comb.

9 – GIST 430 Veh

10 – GIST 430 IM

11 – GIST 430 MK-4440

12 – GIST 430 Comb.

Marker's size map:

Sizes used for MOPS buffer

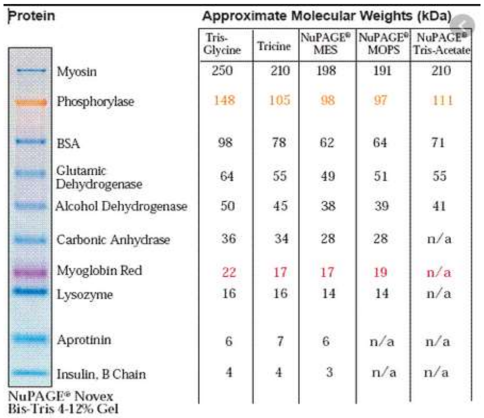

Protein	Tris-Glycine	Tricine	NuPAGE® MES	NuPAGE® MOPS	NuPAGE® Tris-Acetate
Myosin	250	210	198	191	210
Phosphorylase	148	105	98	97	111
BSA	98	78	62	64	71
Glutamic Dehydrogenase	64	55	49	51	55
Alcohol Dehydrogenase	50	45	38	39	41
Carbonic Anhydrase	36	34	28	28	n/a
Myoglobin Red	22	17	17	19	n/a
Lysozyme	16	16	14	14	n/a
Aprotinin	6	7	6	n/a	n/a
Insulin, B Chain	4	4	3	n/a	n/a

Approximate Molecular Weights (kDa)

NuPAGE® Novex Bis-Tris 4-12% Gel

pKIT (120/145kDa)

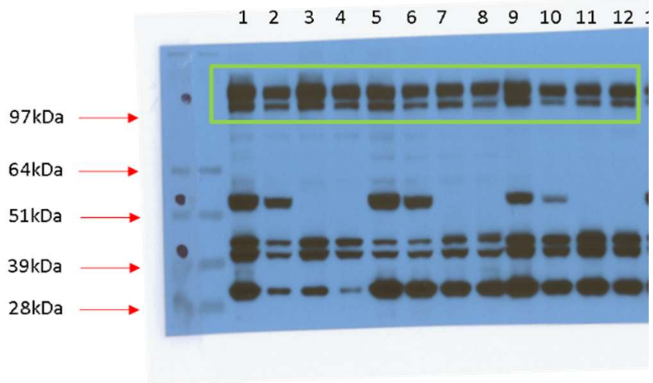

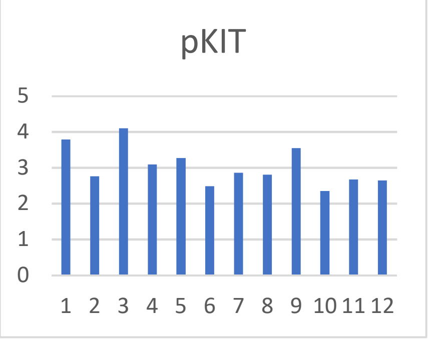

KIT (120/145kDa)

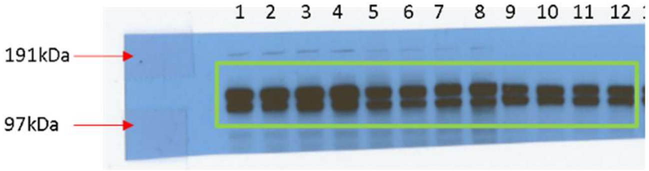

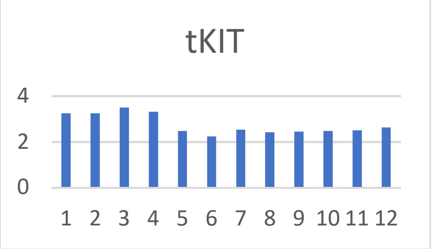

pAKT (60kDa)

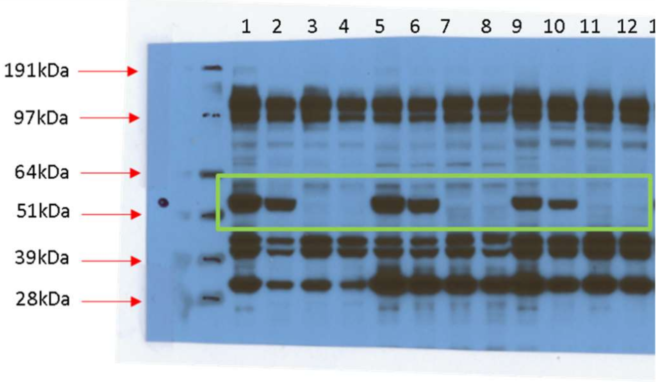

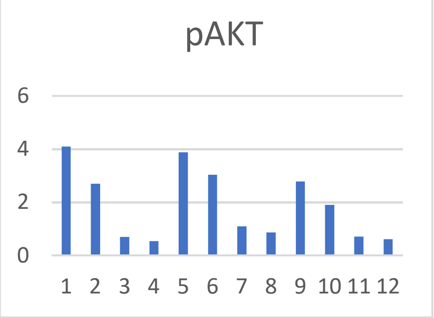

AKT (60kDA)

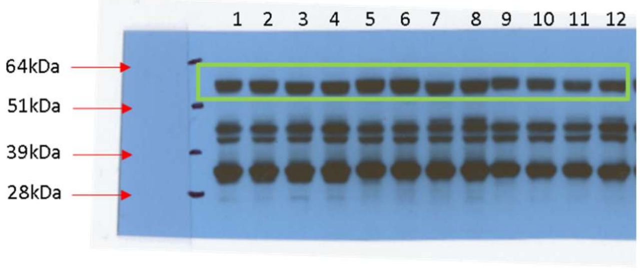

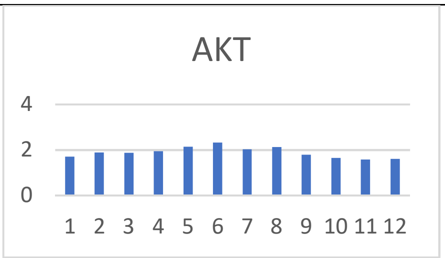

pMAPK (42/44kDa)

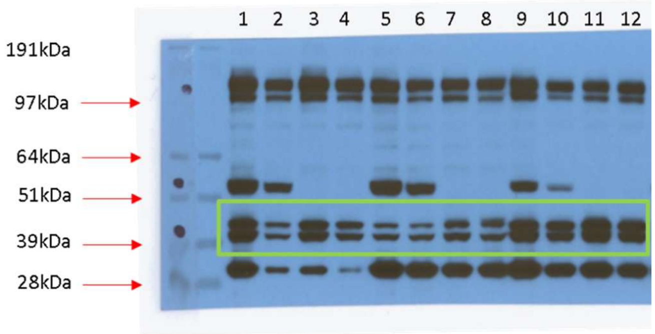

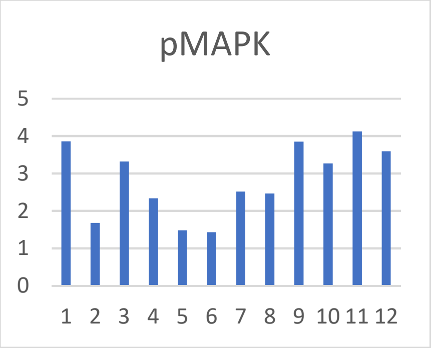

MAPK (42/44kDa)

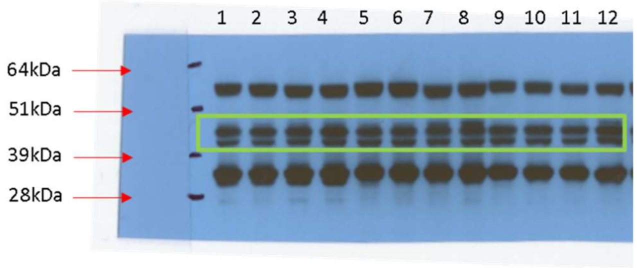

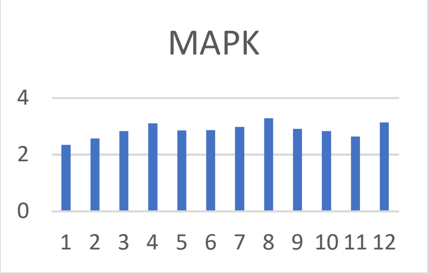

pS6 (32kDa)

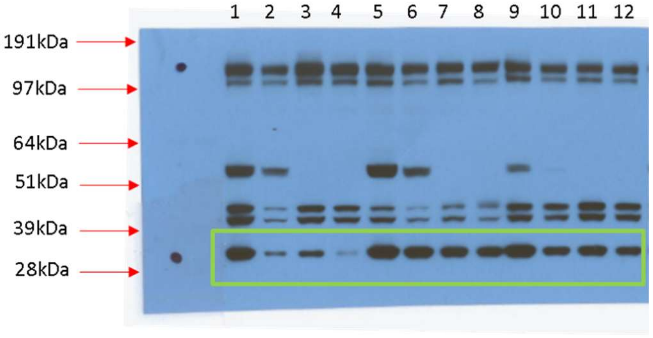

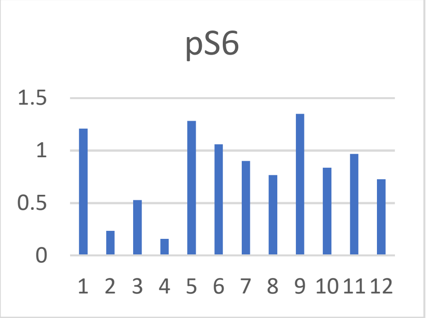

S6 (32kDa)

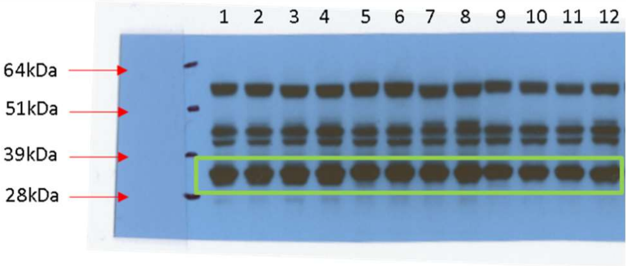

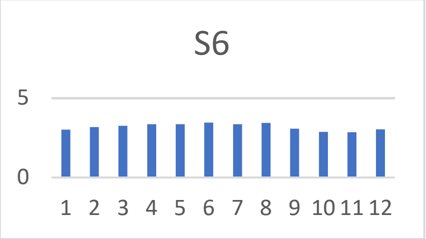

GAPDH (37kDa)

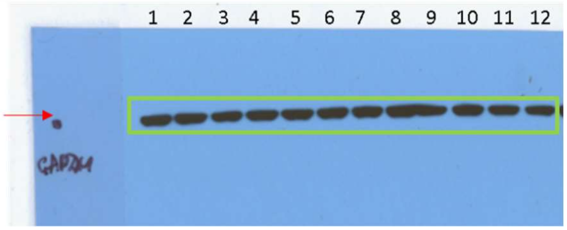

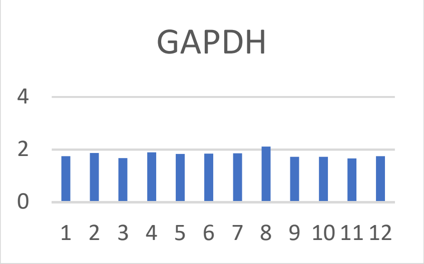

Figure 4

Samples' naming:

1 – GIST-T1 Veh

2 – GIST-T1 IM

3 – GIST-T1 MK-4440

4 – GIST-T1 Comb.

5 – GIST-T1/829 Veh

6 – GIST-T1/829 IM

7 – GIST-T1/829 MK-4440

8 – GIST-T1/829 Comb.

9 – GIST 430 Veh

10 – GIST 430 IM

11 – GIST 430 MK-4440

12 – GIST 430 Comb.

Marker's size map:

Sizes used for MOPS buffer

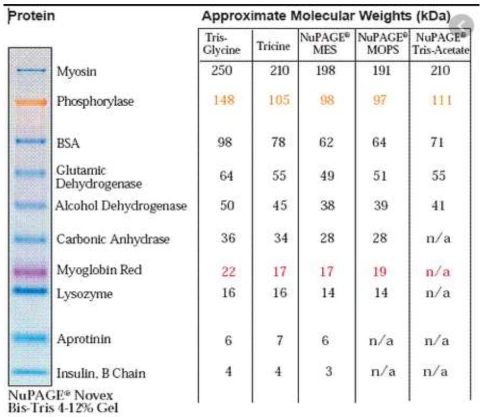

Protein	Approximate Molecular Weights (kDa)				
	Tris-Glycine	Tricine	NuPAGE® MES	NuPAGE® MOPS	NuPAGE® Tris-Acetate
Myosin	250	210	198	191	210
Phosphorylase	148	105	98	97	111
BSA	98	78	62	64	71
Glutamic Dehydrogenase	64	55	49	51	55
Alcohol Dehydrogenase	50	45	38	39	41
Carbonic Anhydrase	36	34	28	28	n/a
Myoglobin Red	22	17	17	19	n/a
Lysozyme	16	16	14	14	n/a
Aprotinin	6	7	6	n/a	n/a
Insulin, B Chain	4	4	3	n/a	n/a

NuPAGE® Novex Bis-Tris 4-12% Gel

PDCD4 (60kDa)

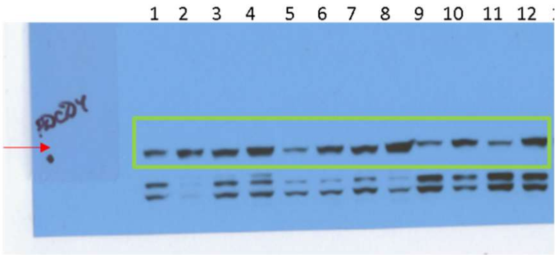

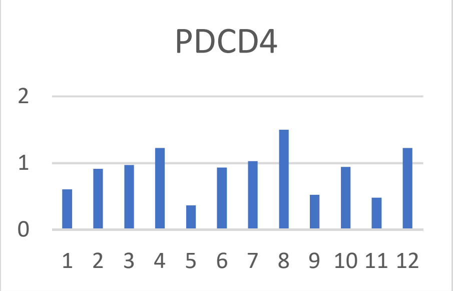

PARP1 (116kDa)
Cleaved PARP (89kDa)

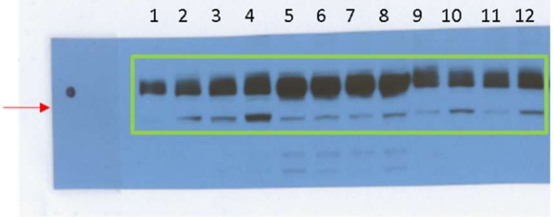

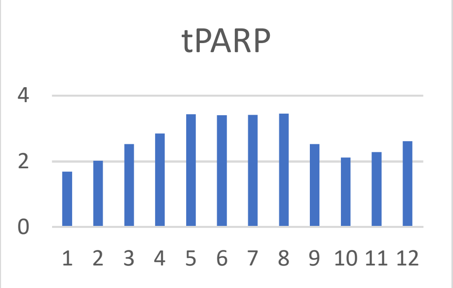

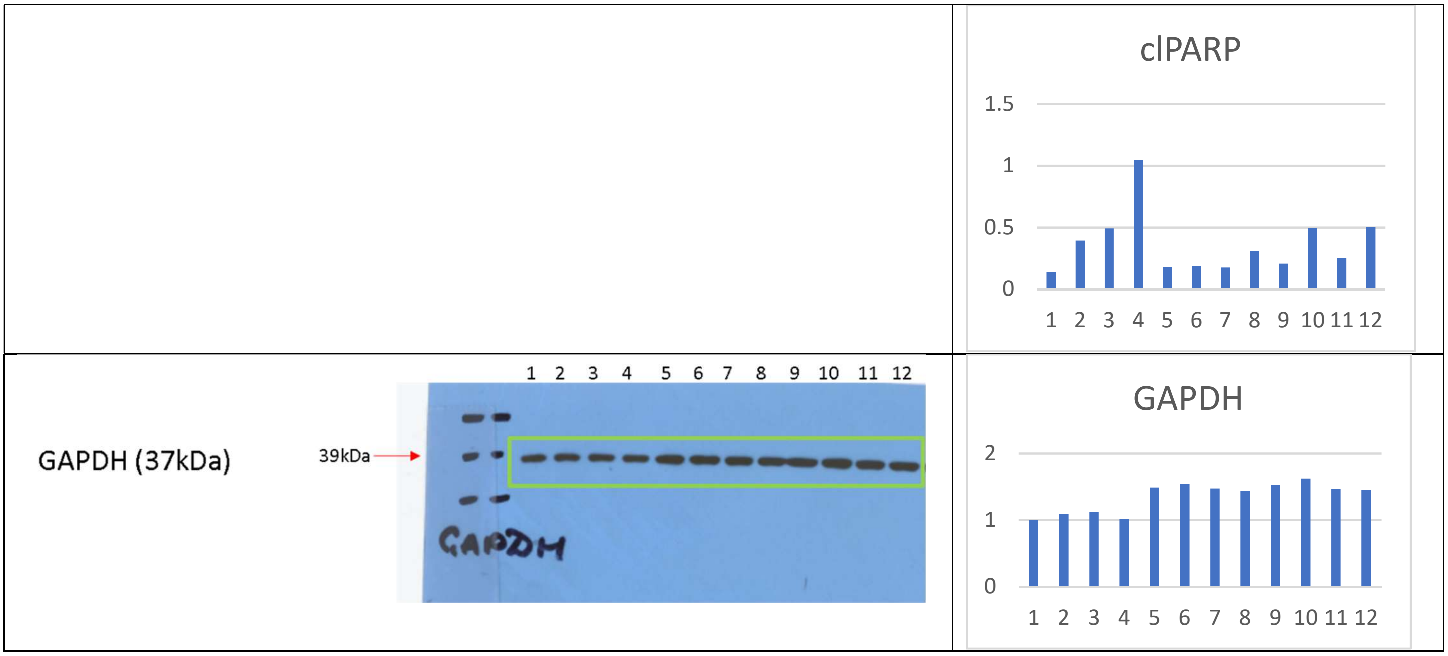

Supplementary Figure 1

Samples' naming:

1 – GIST-T1 Control

2 – GIST-T1 PDCD4 overexpression

3 – GIST430 Control

4 – GIST430 PDCD4 overexpression

5 – GIST-T1/829 Control

6 – GIST-T1/829 PDCD4 overexpression

Marker's size map:

Sizes used for MOPS buffer

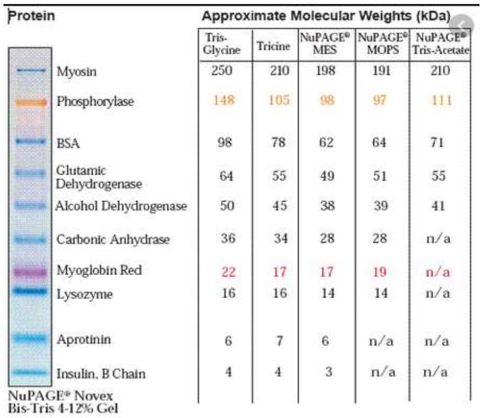

Protein	Approximate Molecular Weights (kDa)				
	Tris-Glycine	Tricine	NuPAGE® MES	NuPAGE® MOPS	NuPAGE® Tris-Acetate
Myosin	250	210	198	191	210
Phosphorylase	148	105	98	97	111
BSA	98	78	62	64	71
Glutamic Dehydrogenase	64	55	49	51	55
Alcohol Dehydrogenase	50	45	38	39	41
Carbonic Anhydrase	36	34	28	28	n/a
Myoglobin Red	22	17	17	19	n/a
Lysozyme	16	16	14	14	n/a
Aprotinin	6	7	6	n/a	n/a
Insulin, B Chain	4	4	3	n/a	n/a

NuPAGE® Novex Bis-Tris 4-12% Gel

PDCD4 (60kDa) 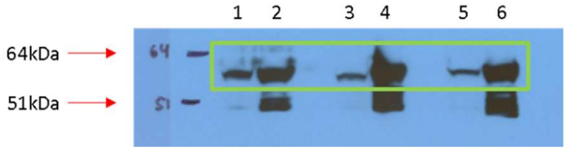	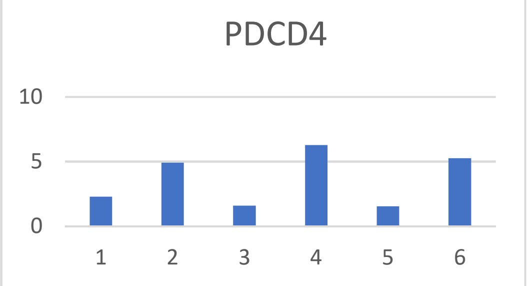
PARP1 (116kDa)<br>Cleaved PARP (89kDa) 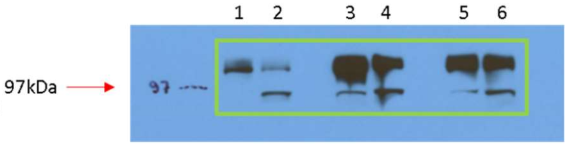	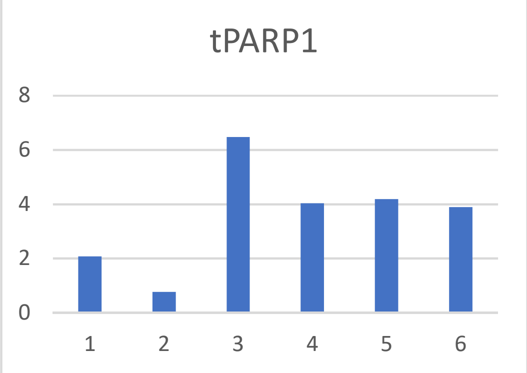
	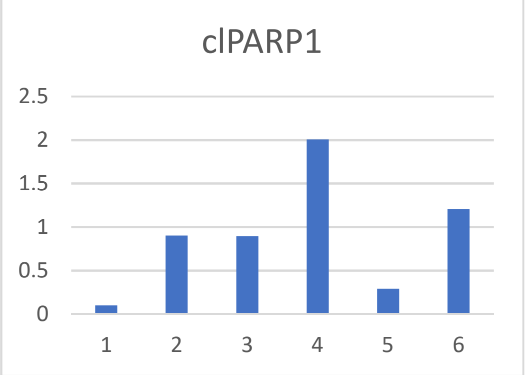
P27 (27 kDa) 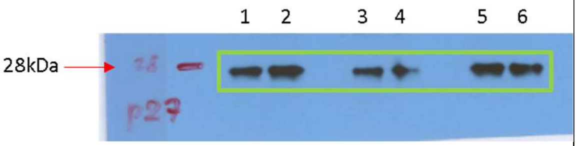	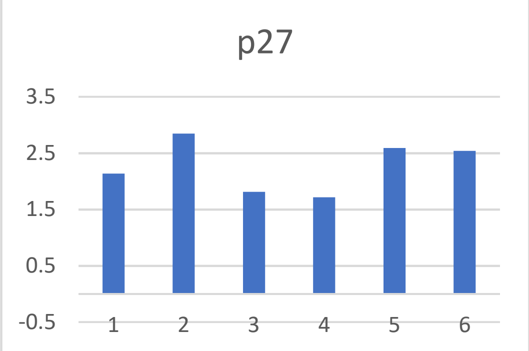
GAPDH (37kDa) 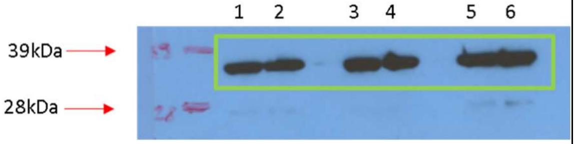	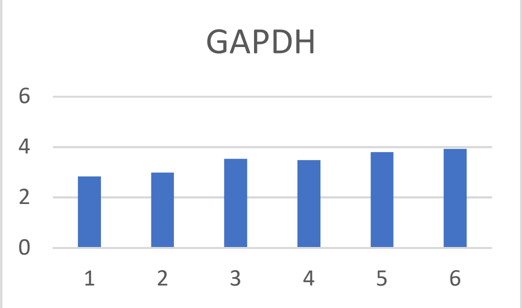

Supplementary Figure 2

Samples' naming:

1 – GIST-T1 Veh

2 – GIST-T1 Ripretinib

3 – GIST-T1 MK-4440

4 – GIST-T1 Comb.

5 – GIST-T1/829 Veh

6 – GIST-T1/829 Ripretinib

7 – GIST-T1/829 MK-4440

8 – GIST-T1/829 Comb.

9 – GIST 430 Veh

10 – GIST 430 Ripretinib

11 – GIST 430 MK-4440

12 – GIST 430 Comb.

Marker's size map:

Sizes used for MOPS buffer

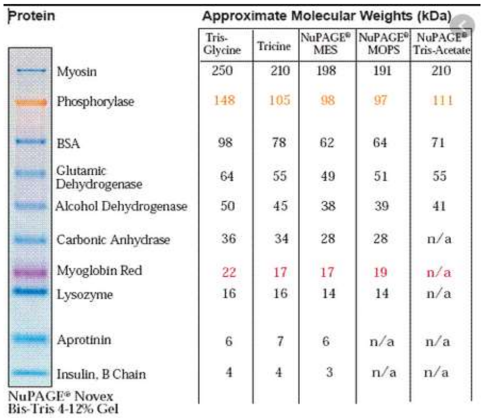

Protein	Approximate Molecular Weights (kDa)				
	Tris-Glycine	Tricine	NuPAGE® MES	NuPAGE® MOPS	NuPAGE® Tris-Acetate
Myosin	250	210	198	191	210
Phosphorylase	148	105	98	97	111
BSA	98	78	62	64	71
Glutamic Dehydrogenase	64	55	49	51	55
Alcohol Dehydrogenase	50	45	38	39	41
Carbonic Anhydrase	36	34	28	28	n/a
Myoglobin Red	22	17	17	19	n/a
Lysozyme	16	16	14	14	n/a
Aprotinin	6	7	6	n/a	n/a
Insulin, B Chain	4	4	3	n/a	n/a

NuPAGE® Novex Bis-Tris 4-12% Gel

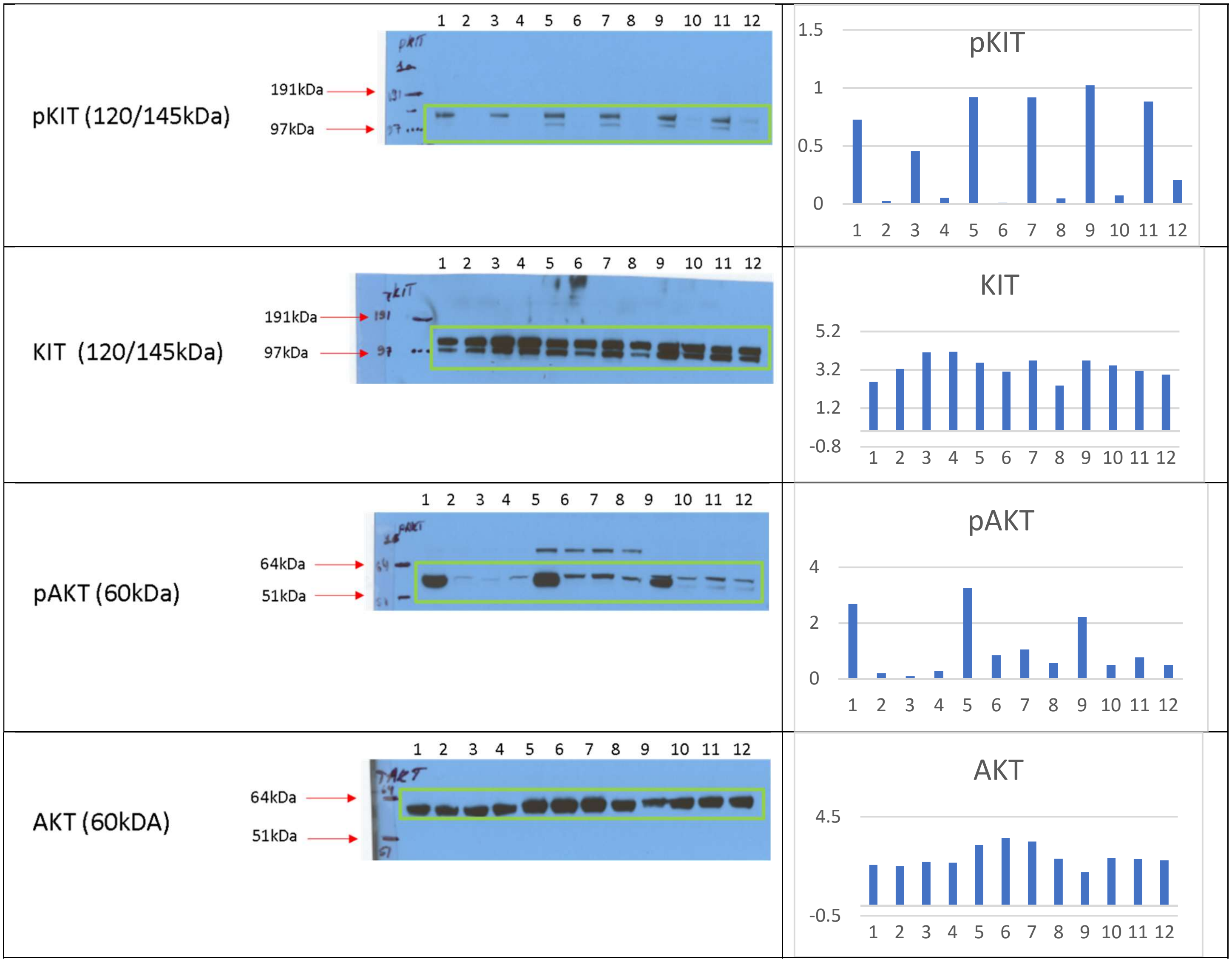

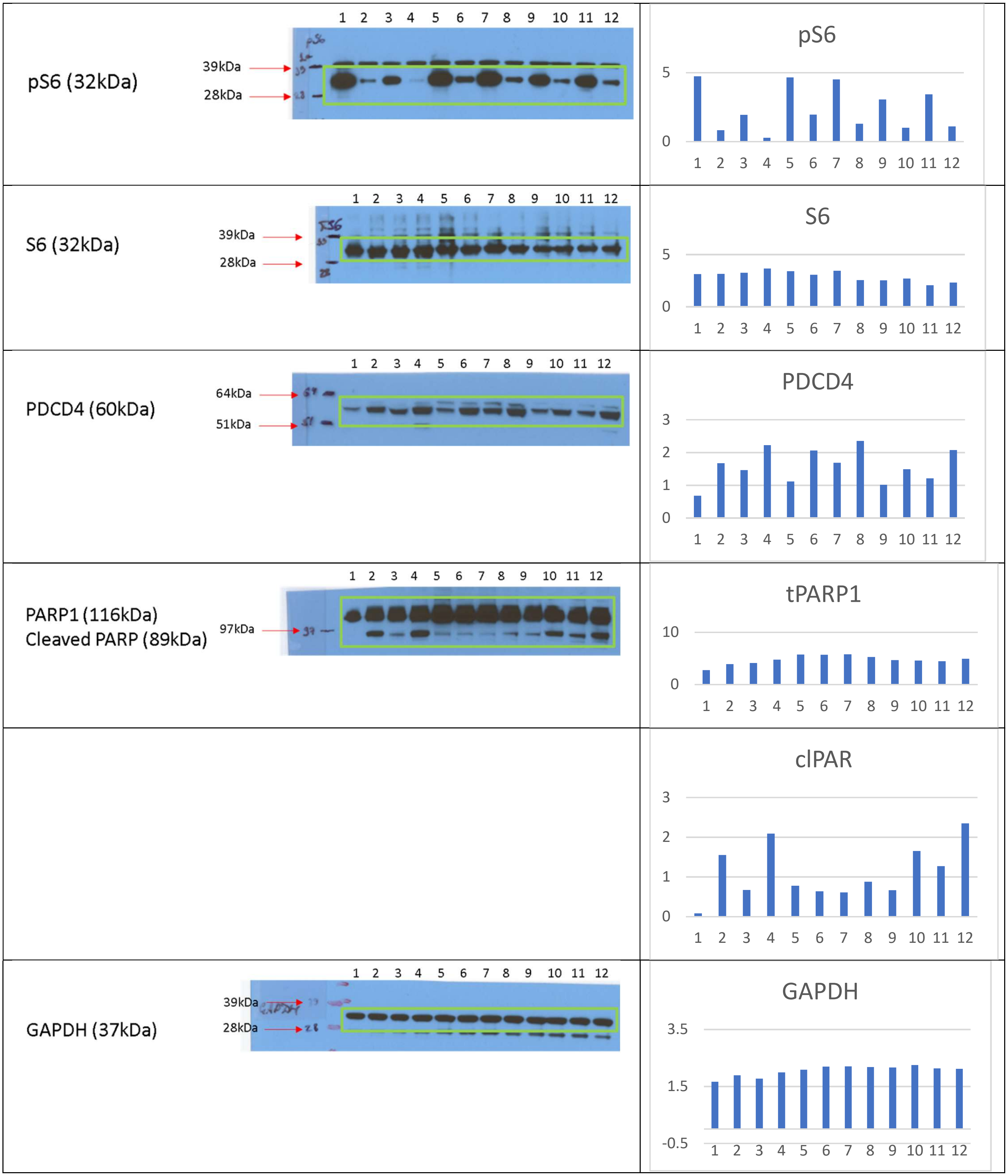
pS6 (32kDa)
39kDa
28kDa
1 2 3 4 5 6 7 8 9 10 11 12
pS6
5
0
S6 (32kDa)
39kDa
28kDa
S6
PDCD4 (60kDa)
64kDa
51kDa
PDCD4
3
2
1
PARP1 (116kDa)
Cleaved PARP (89kDa)
97kDa
tPARP1
10
clPAR
GAPDH (37kDa)
GAPDH
3.5
1.5
-0.5
